# Extraskeletal Chondrosarcoma of Labium Majus

**DOI:** 10.1155/2011/429562

**Published:** 2011-09-15

**Authors:** Arshad S. Khan, Girish D. Bakhshi, Aftab Shaikh, Ashraf A. Khan, Adil A. Khan, Arun Chitale

**Affiliations:** ^1^Department of Surgery, Grant Medical College & Sir J. J. Group of Hospitals, Mumbai 400008, India; ^2^Plot-61, Sector-7, Koper-Khairane, New Mumbai-400709, Maharashtra, India; ^3^Department of Pathology, Bombay Hospital, Mumbai 400020, India

## Abstract

Extraskeletal myxoid chondrosarcoma (ESMC) is a rare tumor seen more often in men. It is seen to arise from soft tissue of lower extremity or buttocks. We report a case of soft tissue swelling of left labium majus in a 66-year-old female. Patient underwent wide excision with uneventful postoperative course. Histopathology of specimen confirmed it to be ESMC. Patient refused adjuvant therapy. Followup of 1 year has shown her to be disease- and symptom- free. Only two cases arising from vulva have been reported in literature . This is the third case and first from Indian subcontinent. A brief review of clinical features, diagnosis, treatment, and outcome of patients with extraskeletal chondrosarcoma is presented.

## 1. Introduction

Extraskeletal myxoid chondrosarcoma (ESMC) was first described by Enzinger and Shiraki [1–3]. Its ultrastructural and molecular features are distinct from skeletal myxoid chondrosarcoma [1]. It is a slowly growing tumor which rarely has metastases, however it is prone to local recurrences. [1]. It has been reported to arise from extremities, neck orbit, and genitourinary tract [2–4]. This tumor presents as a painless lump in majority of cases, and preoperative diagnosis may be difficult.

We present a case of ESMC arising from left labium majus in a 66-year-old female. Surgical excision followed by histopathology confirmed the diagnosis. 

## 2. Case Report

**Figure 1:**
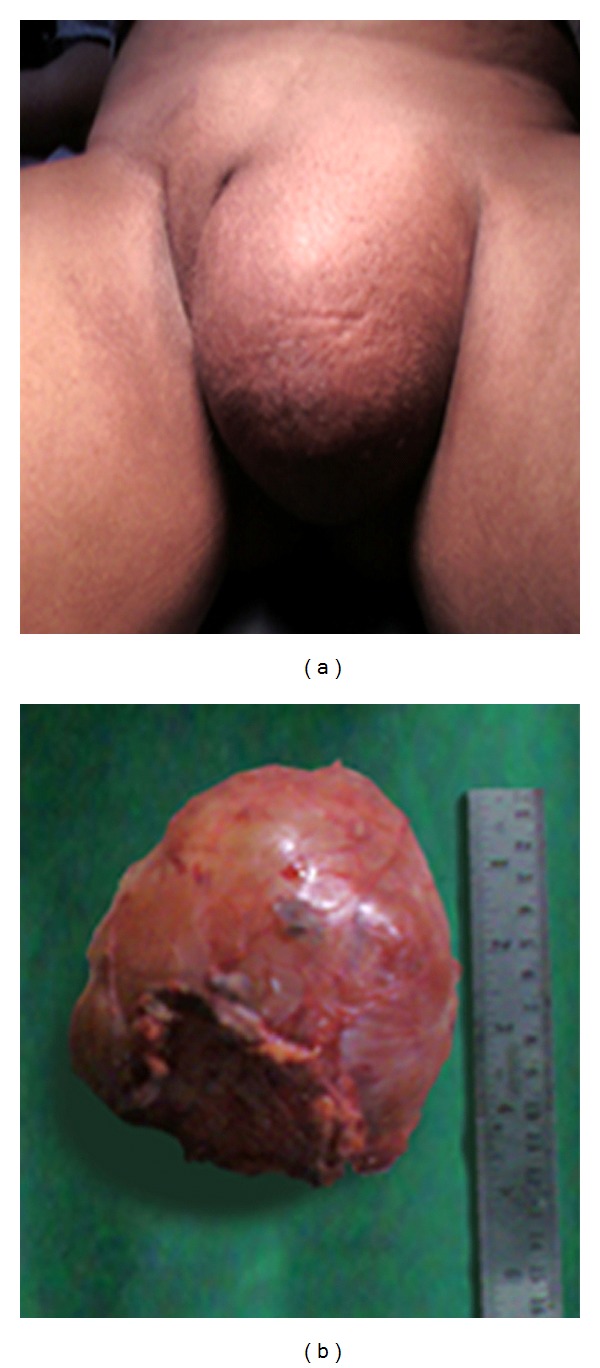
(a) Preoperative picture showing soft tissue mass arising within left labium major. (b) Encapsulated tumor after excision.

**Figure 2:**
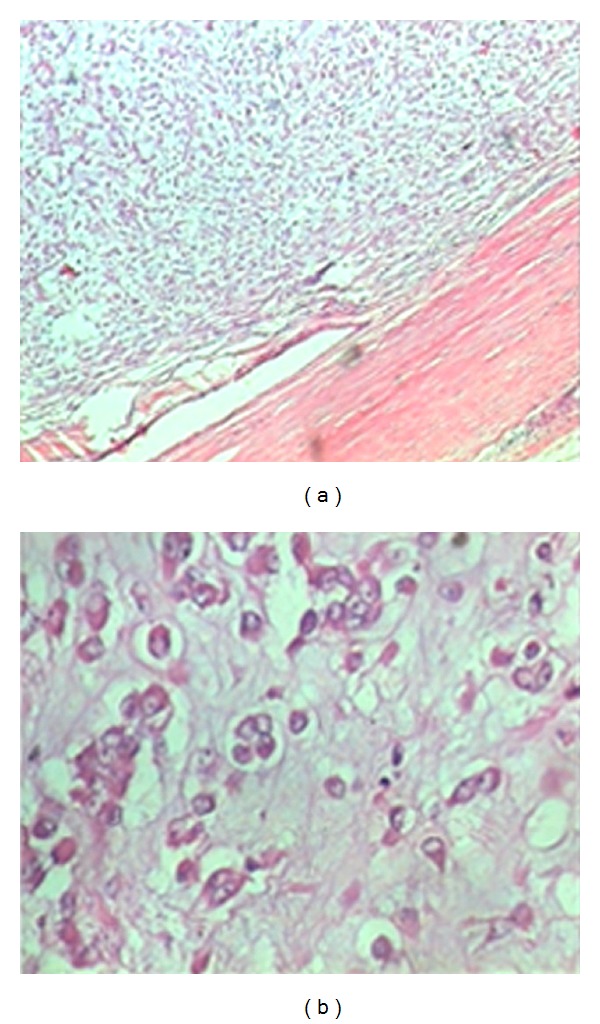
(a) Encapsulated sarcomatous tumor (Hematoxylin & Eosin stain 80X). (b) Single and small groups of vacuolated cells separated by abundant mucoid matrix (Hematoxylin & Eosin stain 200X).

 A 66-year-old female presented with painless lump in the left vulvar region for 2 years. It was initially small and had gradually increased in size. There was no history of fever, trauma, or any other complaints. She has been post menopausal for 20 years. Clinical examination revealed a firm, nontender lump measuring 12 cms × 8 cms arising from the left labium major with smooth surface and well-defined margins (Figure 1(a)). Ultrasonography (USG) revealed it to be a soft tissue mass with cystic areas involving left labium majus. There was no inguinal lymphadenopathy. Fine needle aspiration cytology (FNAC) was inconclusive. Exploration through a longitudinal incision revealed a tumor above the adductor tendon but not fixed to it. Tumor was dissected out completely and excised (Figure 1(b)). Incision closed with a drain. Postoperative course was uneventful. Histopathology of the specimen showed a soft tissue sarcoma enveloped in a thick complete fibrous capsule (Figure 2(a)). It consisted of lobules of oval or slightly elongated cells in rows, strands and sheets with intervening myxomatous stroma.The cells possessed deeply eosinophilic dense cytoplasm and large oval vesicular nuclei with nucleoli (Figure 2(b)). Mitosis was rare. The amount of mucoid matrix varied from area to area but was in excess in some parts.There was no capsular or vascular invasion. Hence, final diagnosis of myxoid chondrosarcoma was made. Patient refused adjuvant therapy. Followup of 1 year has shown her to be disease- and symptom- free.

## 3. Discussion

ESMC is a rare variety of sarcoma with a cytogenetic basis. The EWS-CHN gene fusion is seen in more than 75% cases of ESMC [5]. It is seen more often in the lower extremities and buttocks, however case reports of ESMC of orbit, shoulder, and upper extremity have been reported. This is seen more commonly in men and often asymptomatic [1]. Our case was a 66-year-old female with lump arising from labium majus.

Preoperative diagnosis is difficult, however there are studies where fine needle aspiration cytology (FNAC) has diagnosed this entity preoperatively [6]. 

Microscopically, extraskeletal myxoid chondrosarcoma has a multinodular pattern. The tumor nodules consist of round or slightly elongated cells of uniform shape and size in variable amounts of mucoid material. The neoplastic cells are arranged in short anastomosing cords or strands, and less frequently in small, loose aggregates. The individual cells have small hyperchromatic nuclei and a narrow rim of deeply eosinophilic cytoplasm and feature characteristic of chondroblasts [7]. Our case histopathology of the excised specimen confirmed the diagnosis. Immunohistochemistry studies have shown that the tumor cells usually express vimentin, synaptophysin, S-100 protein, and epithelial membrane antigen [8]. Prognostic factors like, tumor size ≥10 cm, high cellularity, presence of anaplasia, and a mitotic activity of more than two per ten high power fields are associated with decreased survival [8]. However, in our case inspite of large size, the prognosis has been good after excision. 

The treatment of patients with localized extraskeletal myxoid chondrosarcoma should include excision of the primary tumor with a wide surgical margin [9]. ESMC has been reported to respond to high-dose radiation while being poorly responsive to adjuvant chemotherapy. When a wide margin cannot be obtained, marginal surgery with adjuvant radiation can be one of the options for treatment [9]. In our case, wide excision was done with no adjuvant therapy as the patient refused adjuvant therapy. Our case compels us to think that only wide excision may be the only modality of treatment in certain cases, however factors governing this have to be explored by studying more cases.
